# Perceptions and Understandings of Frailty Language: A Scoping Review

**DOI:** 10.1093/geroni/igaa057.572

**Published:** 2020-12-16

**Authors:** Mariko Sakamoto, Pamela Durepos, Kyla Alsbury, Patricia Hewston, Alyson Takaoka, Julia Borges

**Affiliations:** 1 University of British Columbia, Vancouver, British Columbia, Canada; 2 McMaster University, Hamilton, Ontario, Canada; 3 University of Toronto, Toronto, Ontario, Canada; 4 Dalhousie University, Halifax, Nova Scotia, Canada

## Abstract

Diagnosing and responding to frailty in older adult populations is of growing interest for health care professionals, researchers and policymakers. Preventing frailty has the potential to improve health outcomes for older adults, which in turn has significant implications for health care systems. However, little is known about how older people understand and perceive the term “frailty”, and what it means for them to be designated as frail. To address this concern, a scoping review was undertaken to map the breadth of primary research studies that focus on community-dwelling older adults’ perceptions and understanding of frailty language, as well as explore the potential implications of being classified as frail. Searches were conducted in MEDLINE, Ageline, PsychInfo, CINAHL and EMBASE databases for articles published between January 1994 and February 2019. 4639 articles were screened and ten articles met the inclusion criteria, detailing eight primary research studies. Using content analysis, three core themes were identified across the included studies. These themes included: 1) understanding frailty as a multi-dimensional concept and inevitable consequence of aging, 2) perceiving frailty as a generalizing and harmful label; and 3) resisting and responding to frailty. Recommendations stemming from this review include the need for health care professionals to use person-centered language with older adults, discuss the term frailty with caution, and be aware of the potential consequences of labeling a person as frail. Importantly, this review demonstrates that for frailty interventions to be successful and meaningful for older adults, ongoing and critical examination of frailty language is necessary.

